# Blockade of the PD-1/PD-L1 Immune Checkpoint Pathway Improves Infection Outcomes and Enhances Fungicidal Host Defense in a Murine Model of Invasive Pulmonary Mucormycosis

**DOI:** 10.3389/fimmu.2022.838344

**Published:** 2022-02-18

**Authors:** Sebastian Wurster, Nathaniel D. Albert, Uddalak Bharadwaj, Moses M. Kasembeli, Jeffrey J. Tarrand, Naval Daver, Dimitrios P. Kontoyiannis

**Affiliations:** ^1^ Department of Infectious Diseases, Infection Control and Employee Health, The University of Texas MD Anderson Cancer Center, Houston, TX, United States; ^2^ Department of Laboratory Medicine, The University of Texas MD Anderson Cancer Center, Houston, TX, United States; ^3^ Department of Leukemia, The University of Texas MD Anderson Cancer Center, Houston, TX, United States

**Keywords:** mucormycosis, animal model, checkpoint inhibitors, immunotherapy, T cells, cytokines

## Abstract

Anecdotal clinical reports suggested a benefit of adjunct immune checkpoint inhibitors (ICIs) to treat invasive mucormycosis. However, proof-of-concept data in animal models and mechanistic insights into the effects of ICIs on host defense against Mucorales are lacking. Therefore, we studied the effects of PD-1 and PD-L1 inhibitors (4 doses of 250 µg/kg) on outcomes and immunopathology of invasive pulmonary mucormycosis (IPM) in cyclophosphamide- and cortisone acetate-immunosuppressed mice. *Rhizopus arrhizus*-infected mice receiving either of the ICI treatments had significantly improved survival, less morbidity, and lower fungal burden compared to isotype-treated infected mice. While early improvement of morbidity/mortality was comparable between the ICI treatments, anti-PD-L1 provided more consistent sustained protection through day 7 post-infection than anti-PD-1. Both ICIs enhanced the fungicidal activity of *ex-vivo* splenocytes and effectively counteracted T-cell exhaustion; however, macrophages of ICI-treated mice showed compensatory upregulation of other checkpoint markers. Anti-PD-1 elicited stronger pulmonary release of proinflammatory cytokines and chemokines than anti-PD-L1, but also induced cytokines associated with potentially unfavorable type 2 T-helper-cell and regulatory T-cell responses. Although no signs of hyperinflammatory toxicity were observed, mice with IPM receiving ICIs, particularly anti-PD-1, had elevated serum levels of IL-6, a cytokine linked to ICI toxicities. Altogether, inhibition of the PD-1/PD-L1 pathway improved clinical outcomes of IPM in immunosuppressed mice, even without concomitant antifungals. PD-L1 inhibition yielded more favorable immune responses and more consistent protection from IPM-associated morbidity and mortality than PD-1 blockade. Future dose-effect studies are needed to define the “sweet spot” between ICI-induced augmentation of antifungal immunity and potential immunotoxicities.

## Introduction

Invasive mucormycosis (IM) is an aggressive and frequently lethal mold infection (1, 2). Pneumonia is the most common manifestation of IM in patients with severe immunosuppression (1, 2). As immunological recovery is a major determinant of therapeutic success (3, 4), facile adjunct immune enhancement strategies are a major unmet need to improve the detrimental outcomes of invasive mold infections (5, 6). Although cellular immune therapeutics such as adoptive T-cell transfer yielded promising results *in-vitro* and in animal models of IM (7–9), the clinical translation of these approaches is hampered by feasibility concerns (e.g., time-consuming production of cellular products), high cost, and regulatory obstacles (3, 5, 10).

Unlike cell-based immunotherapies, immune checkpoint inhibitors (ICIs) could provide a readily available tool to augment host defense against opportunistic pathogens, including molds (11, 12). Pathogenic molds were shown to induce checkpoint pathways that can drive immune exhaustion and impair fungal clearance (13, 14). Consequently, several *in-vivo* studies and clinical case reports suggested a potential of ICIs, especially agents blocking the Programmed Cell Death Protein 1 (PD-1) pathway, as an adjunct treatment for invasive mold infections (14–18). However, no systematic preclinical studies exist regarding the impact of ICIs on the immunopathology and outcomes of IM. Furthermore, studies directly comparing blockade of PD-1 or its ligand PD-L1 in mammalian models of invasive mold infections are lacking.

Therefore, we herein compared infection outcomes and key immune parameters in pharmacologically immunosuppressed mice with invasive pulmonary mucormycosis (IPM) that were treated with PD-1 or PD-L1 inhibitors versus non-targeting isotype antibodies. We found that monotherapy with PD-1/PD-L1 inhibitors significantly improved morbidity and mortality of IPM. We further defined immune features that were linked to ICI-mediated protection and potential ICI toxicity in mice with IPM, particularly focusing on disparate cytokine and chemokine responses elicited by PD-1 versus PD-L1 blockade.

## Materials and Methods

### Preparation of Fungal Inoculum

*Rhizopus arrhizus* Ra-749 (clinical isolate) was cultured on Sabouraud dextrose agar plates for 2 days at 37°C. The mycelium was overlaid with sterile saline and spores were collected by gently scraping the mycelium with a sterile glass rod. The spore suspension was washed twice with sterile saline and spore concentrations were determined with a hemocytometer. For live imaging studies, an FTR1-GFP *R. arrhizus* mutant was cultured on uracil-deficient selection medium, as previously described (19).

### Murine Infection Model and Treatments

**Figure 1:**
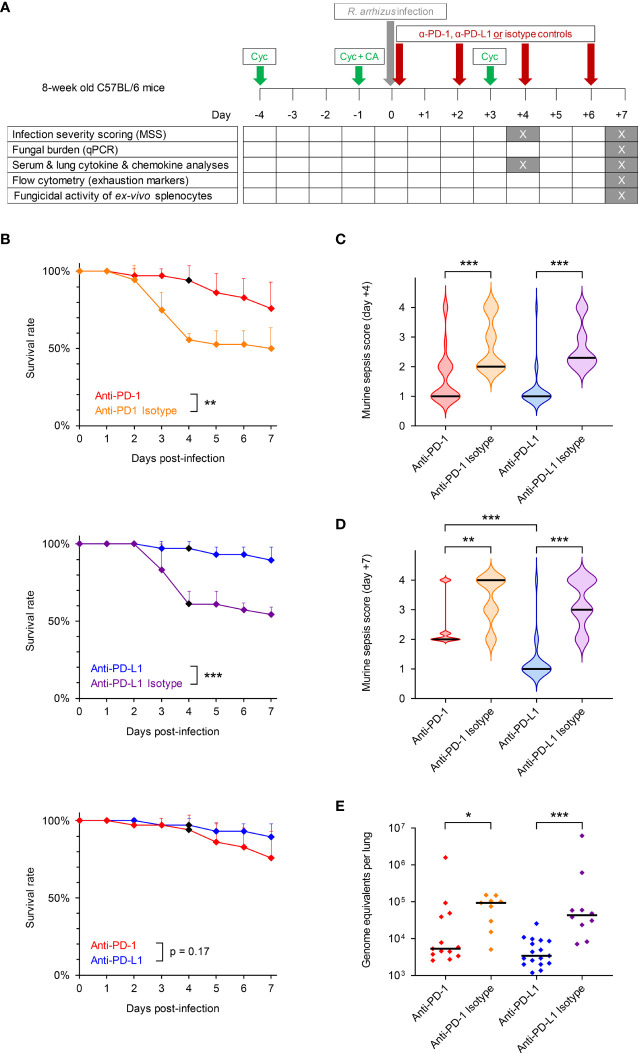
Inhibition of the PD-1/PD-L1 immune checkpoint pathway improves infection outcomes in immunosuppressed mice with invasive pulmonary mucormycosis (IPM). **(A)** Timeline of experimental procedures. Eight-week-old female C57BL/6 mice were immunosuppressed with cyclophosphamide (Cyc) and cortisone acetate (CA) and were subsequently infected intranasally with 50,000 spores of *R. arrhizus* Ra-749, as described in Materials & Methods. Mice then received 4 intraperitoneal injections of anti-PD-1, anti-PD-L1, or the corresponding unspecific isotype control antibodies on days 0 (6 h post-infection), +2, +4, and +6. Survival was monitored for 7 days post-infection and morbidity was scored on days +4 and +7 using the murine sepsis score. Biospecimens for downstream readouts were obtained on days +4 and +7, as detailed in the figure. **(B)** Survival rates of mice with IPM according to the treatment arm (n = 34-36 animals per treatment). Error bars indicate inter-replicate standard deviations based on 3 independent experiments. Animals euthanized in non-moribund condition for biomaterial sampling on day +4 were censored (black diamonds). Mantel-Cox log rank-test. **(C, D)** Violin plots summarizing the distribution of murine sepsis scores on day +4 (C, n = 24-26 per treatment) and day +7 (D, n = 19-26 per treatment). Scale: 0 = healthy to 3 = moribund. A score of 4 denotes animals that died prior to the time of analysis. **(E)** Fungal burden per lung according to the treatment, as determined on day +7 using an 18S quantitative PCR assay (n = 9-16 survivors per treatment from 2 independent experiments). **(C–E)** Black bars indicate medians. The Mann-Whitney U test was used to compare ICI- and isotype-treated cohorts as well as the two ICI treatment cohorts. *p < 0.05, **p < 0.01, ***p < 0.001.

Eight-week-old female BALB/cAnNCrl inbred mice (Charles River Laboratories, weight 20-22 g) were immunosuppressed with 3 intraperitoneal injections of cyclophosphamide (Sigma-Aldrich, 150 mg/kg body weight on days -4 and -1, 100 mg/kg on day +3) and a subcutaneous injection of cortisone acetate (Sigma-Aldrich, 300 mg/kg on day -1), as previously described (16, 20). Mice were then infected intranasally with 50,000 spores of *R. arrhizus*, the most common causative species of IPM in cancer patients (2). On days 0 (6 h after infection), 2, 4, and 6 post-infection, mice received intraperitoneal injections of 250 µg/kg PD-1 or PD-L1 blocking antibodies (Leinco Technologies, anti-PD-1 #P362, anti-PD-L1 #P371). Control cohorts received 250 µg/kg of the corresponding isotype antibodies (Leinco Technologies, IgG2a isotype #I-1177 as control for anti-PD-1, IgG2b isotpye #I-1034 as control for anti-PD-L1). Experimental timelines and sampling of biomaterials for immune readouts (described below) are summarized in **Figure 1A**. All procedures were approved by the MD Anderson Cancer Center Institutional Animal Care and Use Committee (protocol 00001734-RN01).

### Morbidity and Mortality Scoring

Mice were monitored daily and survival outcomes were recorded for 7 days post-infection. We performed 3 independent experiments with a total of 34-36 mice per treatment. Animals euthanized in non-moribund condition to obtain biomaterials for downstream assays were censored. Infection severity was scored on days +4 and +7 using the modified murine sepsis score (MSS, 0 = healthy to 3 = moribund) based on general appearance of the mice, level of consciousness, activity, response to auditory and touch stimuli, appearance of the eyes, and respiration quality, according to published criteria (21). Animals that died prior to the time of assessment received a score of 4.

### Determination of Fungal Burden in Lung Tissue Homogenates by Quantitative PCR

Lung tissue homogenates were prepared from surviving mice on day +7, as described before (16). Total genomic DNA was isolated using the DNeasy Blood & Tissue Kit (Qiagen). Fungal burden was determined by quantitative PCR using Mucorales-specific primers and probes for the 18S rRNA gene (22). Numbers of *R. arrhizus* genome equivalents per lung were interpolated from 6-point standard curves using lung samples from uninfected mice that were spiked with 10-fold serial dilutions of uninucleate Ra-749 spores (10^2^-10^7^ spores per sample).

### Cytokine and Chemokine Assays

Lungs from 5 mice per treatment arm were weighed and snap-frozen on days +4 and +7. After thawing, tissue homogenates were generated as previously described (16). In addition, serum was obtained and processed according to published protocols (16). Cytokine and chemokine concentrations per gram of lung tissue and per mL of serum were determined using a custom-designed 19-plex magnetic Luminex assay (R&D Systems) with the following analytes: GM-CSF, M-CSF, TNF-α, IFN-γ, IL-1β, IL-2, IL-4, IL-5, IL-6, IL-10, IL-13, IL-17A, CCL2 (MCP-1), CCL3 (MIP-1α), CCL4 (MIP-1β), CCL5 (RANTES), CCL7 (MCP-3), CXCL2, and C-reactive protein (CRP). The assay was performed according to the manufacturer’s instructions and analyzed using a Luminex 200 device (Luminex Corporation).

### Preparation of Splenocytes

Spleens were aseptically removed from 6 mice per cohort on day +7. Spleen tissue was homogenized in 1 mL of cold PBS supplemented with 2% fetal bovine serum (FBS, Millipore) using 1.5-mL Biomasher II tubes (Nippi Inc.). Homogenates were passed through a 40-µm cell strainer (Falcon) to generate single cell suspensions. Splenocytes were washed with 5 mL of Roswell Park Memorial Institute medium (RPMI) + 2% FBS and counted with a hemocytometer. Five-hundred-thousand cells per mouse were used for the live imaging assay described in section 2.7. The remainder of the cells was cryopreserved in 80% FBS + 20% dimethyl-sulfoxide and stored at -80°C until further use for flow cytometric analyses.

### Time-Lapse Imaging to Determine the Fungicidal Activity of *Ex-Vivo* Splenocytes

FTR1-GFP *R. arrhizus* spore suspensions were prepared in RPMI + 2% glucose + 10% FBS (immune cell medium) at a concentration of 2×10^3^ spores per mL. Two-hundred spores (100 µL) were seeded per well of a 96-well flat bottom plate (Greiner). Splenocytes were diluted in immune cell medium at concentrations of 1×10^6^/mL, 2×10^5^/mL, and 4×10^4^/mL. One-hundred-µL aliquots of the suspensions, containing 100,000 cells (effector/target ratio [E:T] = 500), 20,000 cells (E:T = 100), and 4,000 cells (E:T = 20), respectively, were added to the spores in the 96-well plate. Each splenocyte sample and E:T ratio was tested in duplicate. Controls (6 wells per plate) consisted of spores and immune cell medium only. Plates were imaged hourly (phase and green channel) for 24 h at 37 °C in the IncuCyte ZOOM time lapse microscopy system (Sartorius) equipped with a 10× PLAN FLUOR objective (Sartorius). Mycelial expansion was quantified using the “Green Channel Neurite Length” NeuroTrack feature, as previously described (23), with a neurite coarse sensitivity of 8, a neurite fine sensitivity of 0.6, and a neurite width of 2 µm. Hyphal length per mm^2^ in co-cultures was normalized to the average hyphal length in “fungus only” control wells.

### Flow Cytometry

Splenocytes were thawed, washed with 10 mL of flow cytometry buffer (PBS + 5% FBS + 2 mM ethylenediaminetetraacetic acid), and counted with a hemocytometer. Per panel, 1×10^6^ cells were labelled with fluorescent antibodies diluted in 100 µL of flow cytometry buffer according to the manufacturer’s protocol. Panel 1: CD3-FITC (Miltenyi Biotec), CD279 (PD-1)-PE (Miltenyi Biotec), CD49b-PE-Vio 770 (Miltenyi Biotec), CD152 (Cytotoxic T-Lymphocyte Associated Protein 4, [CTLA-4])-APC (Miltenyi Biotec), and CD366 (T-cell Immunoglobulin and Mucin-Domain Containing-3 [Tim-3])-Brilliant Violet (BioLegend). Panel 2: CD11b-PE (Miltenyi Biotec), CD274 (PD-L1)-PE/Cyanine7 (BioLegend), CD273 (PD-L2)-APC (Miltenyi Biotec), and CD366 (Tim-3)-Brilliant Violet (BioLegend). After labelling, cells were washed, suspended in 300 µL flow cytometry buffer, and transferred to 5-mL round bottom tubes (Falcon). Fifty thousand viable singlets, as determined by light scatter properties, were analyzed with a Gallios flow cytometer (Beckman Coulter). The percentages of PD-1^+^, CTLA-4^+^, and Tim-3^+^ cells among CD3^+^CD49b^-^ T cells and CD3^-^CD49b^+^ natural killer (NK) cells as well as the percentages of PD-L1^+^, PD-L2^+^, and Tim-3^+^ cells among CD11b^+^ macrophages were determined using FlowJo v10.7.2 (FlowJo LLC).

### Statistical Analysis

Survival data were compared using the Mantel-Cox log-rank test. Morbidity scores, fungal burden, and immunological end points were compared using the Mann-Whitney U test (2-group comparisons) or Kruskal-Wallis test with Dunn’s multiple comparison test (4-group comparisons). For cytokine/chemokine concentrations, Benjamini-Hochberg correction for a false discovery rate (FDR) of 0.2 was applied to compensate for multiple testing. The median-to-median ratio (MMR) was used to descriptively compare immune phenotypes depending on the treatment arm. Spearman’s rank correlation coefficient (ρ) was used for correlation analysis. Statistical analyses and data visualization were performed using GraphPad Prism v9 and Microsoft Excel. Significance levels are denoted by asterisks: * p<0.05, ** p<0.01, *** p<0.001.

## Results

### Infection Outcomes

Immunosuppressed mice with IPM receiving the unspecific isotype antibodies developed severe infection and had 7-day mortality rates of 50% (anti-PD-1 isotype) and 46% (anti-PD-L1 isotype), respectively (**Figure 1B**). Median morbidity scores on day 4 post-infection were 2.0 (anti-PD-1 isotype) and 2.3 (anti-PD-L1 isotype), respectively (**Figure 1C**). Morbidity of isotype-treated mice with IPM continuously increased through day +7, with most animals being in moribund condition or succumbing to *R. arrhizus* infection within 7 days of intranasal challenge (**Figure 1D**).

Both ICI antibodies appeared to be well-tolerated by *R. arrhizus*-infected mice, with no noticeable signs of hyper-inflammatory toxicity or distress after the injections. Blockade of PD-L1 provided a strong survival benefit compared to isotype control, as evidenced by near-universal 7-day survival of infected mice (90%, p<0.001 versus isotype, **Figure 1B**). Similarly, the anti-PD-1 treatment provided a significant survival benefit compared to the corresponding isotype control (p=0.009), with a 7-day survival rate of 76% (**Figure 1B**). The trend toward more favorable survival outcomes of anti-PD-L1-treated versus anti-PD-1-treated infected mice did not reach significance (p=0.17, **Figure 1B**).

Median morbidity scores on day 4 post-infection were 1.0 in both ICI-treated cohorts, indicating a significant improvement of infection severity compared to the isotype controls (p<0.001, **Figure 1C**). Median morbidity scores of anti-PD-L1-treated mice remained at 1.0 by day +7 (p<0.001 versus isotype control), whereas a moderate increase in morbidity to a median score of 2.0 was seen in infected mice receiving anti-PD-1 (p=0.002 versus isotype control, p<0.001 versus anti-PD-L1, **Figure 1D**).

Fungal burden in lung tissue of mice surviving until day +7 was significantly lower in the anti-PD-L1-treated cohort compared to isotype control (3.4k versus 43.2k median genome equivalents per lung, p<0.001, **Figure 1E**). Although significant (5.3k versus 93.4k median genome equivalents per lung, p=0.014), the decrease in pulmonary fungal burden of anti-PD-1-treated mice versus isotype-treated animals was less consistent (**Figure 1E**). Taken together, these results suggest a strong therapeutic benefit of PD-1/PD-L1 axis blockade in immunosuppressed mice with IPM, with more consistent and sustained therapeutic responses in anti-PD-L1-treated mice compared to those receiving anti-PD-1.

### Immunopathology

We then sought to obtain a better understanding of the mechanisms that drive improved infection outcomes in ICI-treated mice and to dissect differences in immune enhancement by anti-PD-1 and anti-PD-L1. Therefore, we performed a spectrum of immunoassays on lung tissue, splenocytes, and serum obtained from *R. arrhizus*-infected mice after treatment with ICIs or isotype antibodies (**Figure 1A**).

**Figure 2:**
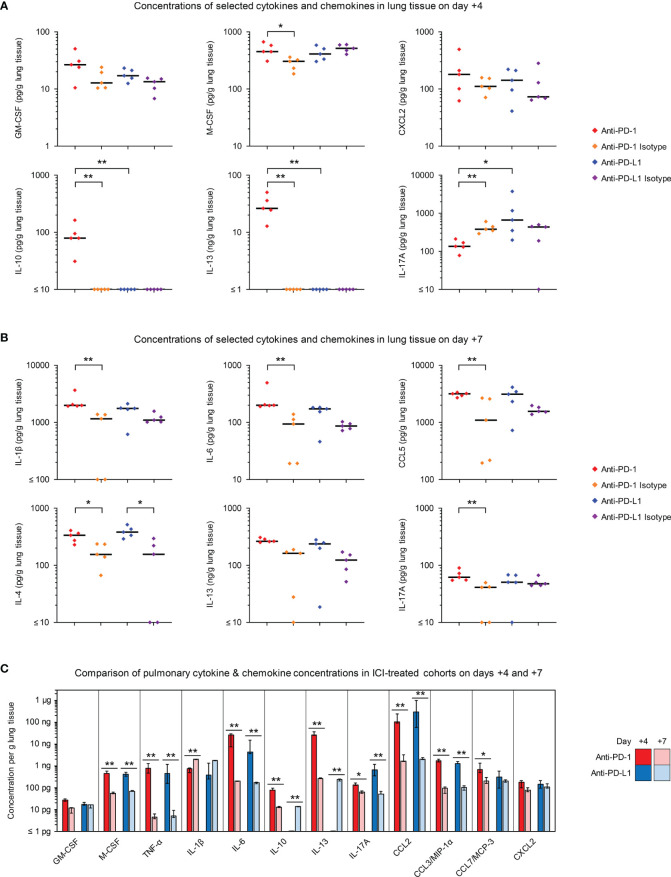
Blockade of PD-1/PD-L1 signaling enhances proinflammatory cytokine release in infected lung tissue. **(A, B)** Cytokine and chemokine concentrations in lung tissue homogenates from *R. arrhizus*-infected mice receiving ICIs or isotype antibodies were measured on day +4 **(A)** and day +7 **(B)** using a 19-plex Luminex assay. Individual and median concentrations of selected cytokines are displayed. Raw data are shown in **Figure S1** (day +4) and **Figure S2** (day +7). The two-sided Mann-Whitney U test with Benjamini-Hochberg adjustment for a false discovery rate of 0.2 was used for significance testing (comparisons between ICI-treated mice and isotype controls as well as between the two ICI-treated cohorts). **(C)** Comparison of median day-4 and day-7 lung concentrations of selected cytokines and chemokines in ICI-treated mice. Error bars represent inter-quartile ranges. The two-sided Mann-Whitney U test with Benjamini-Hochberg adjustment for a false discovery rate of 0.2 was used to compare the two sampling time points for each treatment arm. * p < 0.05, ** p < 0.01. C(X)CL, C(-X)-C motive chemokine receptor ligand; (G)M-CSF, (Granulocyte) Macrophage Colony-Stimulating Factor; IL, Interleukin; MCP, Monocyte Chemoattractant Protein; MIP, Macrophage Inflammatory Protein; TNF, Tumor Necrosis Factor.

Quantification of cytokine concentrations in lung tissue on day +4 revealed elevations of several classical proinflammatory cytokines in mice with IPM receiving anti-PD-1 treatment, including GM-CSF (MMR versus isotype, 2.1), M-CSF (MMR 1.5), IL-6 (MMR 2.7), CCL7 (MMR 2.1), and CXCL2 (MMR 1.6), although significance was only reached for M-CSF due to the limited sample size (**Figures 2A** and **S1**). Blockade of PD-L1 induced similar changes to pulmonary production of proinflammatory cytokines, with modest, non-significant elevations of TNF-α (MMR versus isotype control 2.3), CCL2 (MMR 1.8), CCL7 (MMR 3.2), and CXCL2 (MMR 2.0). Interestingly, mice with IPM receiving the PD-1 inhibitor displayed strongly and significantly elevated production of IL-10 and IL-13, signature cytokines of regulatory T-cells (Treg) and type 2 T-helper (Th2) cells (24). This trend was mirrored by significant suppression of IL-17A, the key cytokine of type 17 T-helper (Th17) cell immunity (24) (**Figures 2A** and **S1**). In contrast, no early changes to pulmonary release of T-cellular signature cytokines were found in anti-PD-L1-treated mice, except for a minor and inconsistent increase in IL-17A compared to isotype-treated mice (MMR 1.5). Direct comparisons of the two ICI-treated cohorts revealed stronger pulmonary production of the proinflammatory cytokines GM-CSF (MMR 1.6), TNF-α (MMR 1.7), IL-6 (MMR 6.1), and CCL7 (MMR 2.2) in anti-PD-1 treated versus anti-PD-L1-treated mice; however, significance was not reached for this observation (**Figures 2A** and **S1**).

Lung concentrations of IL-1β and IL-6 were moderately elevated through day +7 in mice receiving either ICI antibody (**Figures 2B** and **S2**). In addition, elevated levels of CCL5 and cytokines linked to Th2 responses (IL-4, IL-13) were seen in day-7 lung homogenates from both ICI-treated cohorts (**Figures 2B** and **S2**).

Concentrations of most proinflammatory mediators were significantly higher on day +4 than on day +7 in ICI-treated mice (**Figure 2C**). Only IL-1β showed inverse kinetics, whereas GM-CSF and CXCL2 levels were stable over time. Kinetics of IL-10 and IL-13 displayed the strongest disparities between the two ICI-treatments. Day-7 levels of these cytokines were similar in the lungs of anti-PD-L1- and anti-PD-1-treated mice, whereas only the latter group showed strong day-4 release of IL-10 and IL-13 (**Figure 2C**). Altogether, these results suggest modest elevations of proinflammatory cytokines and chemokines in ICI-treated animals with IPM, but also enhancement of cytokine responses that are associated with Th2 and Treg activation, with overall stronger and earlier shifts in anti-PD-1-treated versus anti-PD-1-treated mice.

**Figure 3:**
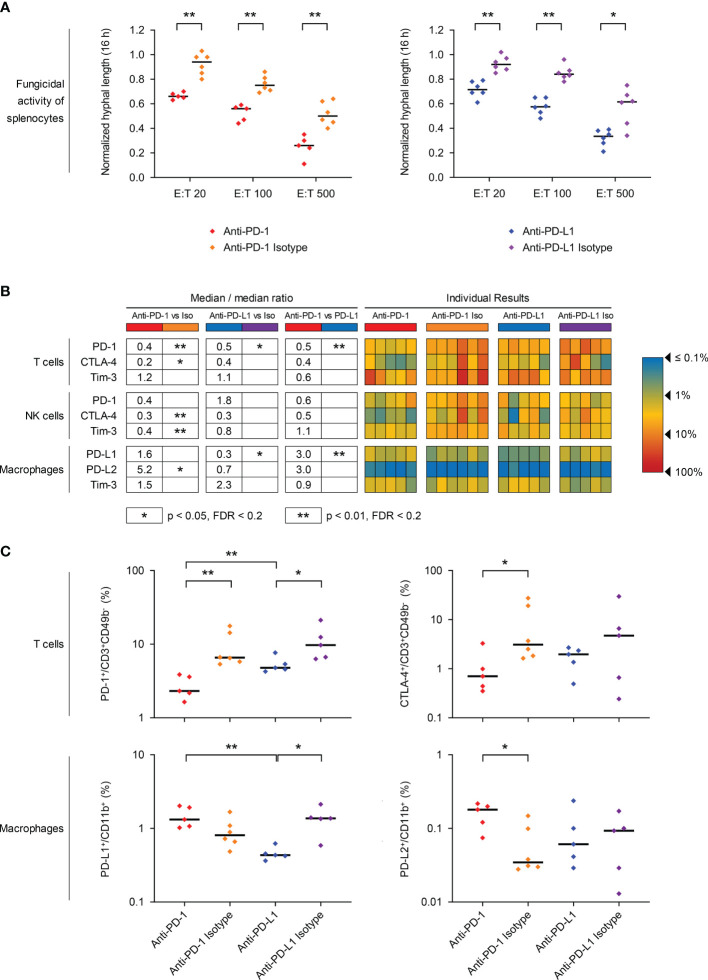
PD-1/PD-L1 axis blockade in mice with invasive pulmonary mucormycosis (IPM) counteracts T-cell exhaustion and enhances the fungicidal activity of *ex vivo* splenocytes. **(A)** *Ex vivo* splenocytes from mice with IPM were co-cultured with a GFP-expressing *R. arrhizus* strain at different effector/target (E:T) ratios and imaged in the IncuCyte time-lapse microscopy system, as described in Materials & Methods. Hyphal length after 16 h of fungus/splenocyte co-culture, normalized to the “fungus only” control (= 1.0), is shown. Individual results for each mouse (n = 5-6 per group) and medians are provided. MMR = median-to-median ration. Mann-Whitney U-test. **(B, C)** Splenic immune cells were isolated from mice with IPM that received ICI treatments or isotype controls and survived the infection for 7 days. The percentages of PD-1, CTLA-4, and Tim-3 expressing T cells (CD3+ CD49b-) and NK cells (CD3-CD49b+) as well as the percentages of PD-L1, PD-L2, and Tim-3 expressing macrophages (CD11b+) were quantified by flow cytometry. **(B)** Heat map summarizing the individual frequencies of checkpoint marker-positive cells. **(C)** Individual and median frequencies (black bars) of selected exhaustion markers are shown. Mann-Whitney U test with Benjamini-Hochberg correction for a false discovery rate of 0.2. * p< 0.05, ** p < 0.01. CD, Cluster of Differentiation; CTLA-4, Cytotoxic T-lymphocyte-Associated Protein 4; MMR, median-to-median ratio; PD-1, Programmed Cell Death Protein 1; PD-L1/2, Programmed Death-Ligand 1/2; Tim-3, T-cell Immunoglobulin and Mucin-Domain Containing-3.

In order to assess the fungicidal activity of *ex-vivo* splenocytes against *R. arrhizus* depending on the treatment received by the mice, we adapted a previously described live imaging assay (23). Across all E:T ratios tested, splenocytes isolated from ICI-treated mice inhibited mycelial proliferation of *R. arrhizus* more potently than cells from animals that received the corresponding isotype antibodies (21-28 percent points reduction in hyphal length, p=0.002-0.015, **Figure 3A**).

Next, we used flow cytometry to analyze the expression of key exhaustion markers on splenic T cells, NK cells, and macrophages 7 days post-infection. The ICI treatments significantly suppressed the expression of their target molecules, as evidenced by strongly reduced frequencies of PD-1-expressing T cells (2.3% *vs*. 6.6%) and NK cells (2.0% *vs*. 4.8%) in anti-PD-1-treated mice and suppression of PD-L1 on macrophages of anti-PD-L1-treated mice (0.4% *vs*. 1.4%) compared to isotype-treated animals (**Figures 3B, C**). Treatment with either of the ICIs also co-suppressed CTLA-4 expression on T cells and NK cells, but moderately induced Tim-3 on macrophages (MMR 1.5-2.9, **Figures 3B, C**). While PD-L2 expression was very low (0.03-0.24%) on macrophages of mice with IPM, regardless of the treatment, significant upregulation was seen in anti-PD-1-treated mice (**Figures 3B, C**). Across all treatment cohorts, PD-1 expression on T-cells (ρ=0.69-0.75, p<0.001) and CTLA-4 expression on NK-cells (ρ=0.54-0.62, p=0.003-0.015) significantly correlated with fungal proliferation in splenocyte co-cultures, suggesting that these markers might be associated with diminished fungicidal activity (**Figure S3**).

**Figure 4:**
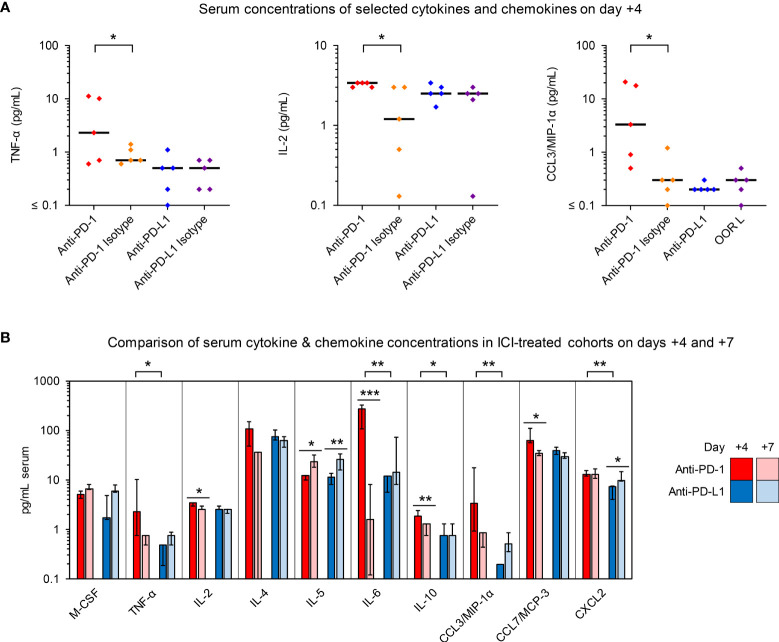
Anti-PD-1- and anti-PD-L1-treated *R. arrhizus*-infected mice display disparate serum kinetics of proinflammatory cytokine and chemokine concentrations. Cytokine and chemokine concentrations in serum samples from *R. arrhizus*-infected mice receiving ICIs or isotype antibodies were quantified using a 19-plex Luminex assay. Raw data are shown in **Figure S4** (day +4) and **Figure S5** (day +7). **(A)** Individual and median concentrations of selected cytokines and chemokines per mL of serum on day +4 (n = 5 mice per treatment arm). **(B)** Comparison of median serum concentrations in anti-PD-1- and anti-PD-L1-treated mice on days +4 (n = 5 mice per treatment arm) and +7 (n = 6-9 mice per treatment arm). Error bars represent inter-quartile ranges. **(A, B)** The two-sided Mann-Whitney U test with Benjamini-Hochberg adjustment for a false discovery rate of 0.2 was used for significance testing. * p< 0.05, ** p < 0.01, *** p < 0.001. C(X)CL, C(-X)-C Motive chemokine receptor ligand; IL, Interleukin; MCP, Monocyte Chemoattractant Protein; M-CSF, Macrophage Colony-Stimulating Factor; MIP, Macrophage Inflammatory Proteins; TNF, Tumor Necrosis Factor.

To further evaluate ICI-mediated changes to the systemic immune environment in mice with IPM, we quantified cytokines and chemokines in serum samples on days +4 and +7. While most cytokine responses were not altered by the ICI treatments, anti-PD-1 elicited significant day-4 elevations of TNF-α, IL-2, and CCL3 compared to mice receiving the isotype control. (**Figures 4A** and **S4**). In direct comparison with anti-PD-L1 therapy, mice receiving the PD-1 inhibitor had significantly stronger elevations of day-4 IL-6 serum levels (median, 271 pg/mL versus 12 pg/mL, p=0.008, **Figures 4B** and **S4**). Although less pronounced, this trend was paralleled by significantly higher day-4 serum levels of TNF-α, IL-10, CCL3, and CXCL2 in anti-PD-1-treated versus anti-PD-L1-treated *R. arrhizus*-infected mice (**Figures 4B** and **S4**). Of note, serum concentrations of these cytokines in individual mice did not correlate with their abundance in lung tissue; therefore, elevated serum concentrations likely do not represent a simple “spillover” effect of lung inflammation (data not shown).

Increased serum levels of proinflammatory cytokines and chemokines in ICI-treated mice with IPM did not persist in animals surviving through day +7 (**Figures 4B** and **S5**). Specifically, median IL-6 serum levels of anti-PD-1-treated mice dropped to 1.6 pg/mL upon discontinuation of ICI treatment (**Figures 4B** and **S5**). Altogether, these results hint at temporary elevations of proinflammatory cytokines in the bloodstream of ICI-treated mice with IPM that are rapidly reversed upon discontinuation of ICI treatment.

## Discussion

ICIs have taken a central stage in modern oncology and an increasing number of studies suggest that ICIs can also improve the outcome of a variety of opportunistic infections, including mucormycosis, in severely immunocompromised patients (11, 12, 14, 15). In order to obtain a better understanding of the immunomodulatory effects of different PD-1 pathway inhibitors, we herein compared infection outcomes and immune features of immunosuppressed mice with IPM after (mono)therapy with PD-1/PD-L1 blocking antibodies or non-targeting isotype antibodies.

We and others previously showed dose-dependently improved outcomes of ICI therapy in murine models of fungal sepsis and invasive pulmonary aspergillosis (IPA), with a stronger benefit of low-dose ICI regimens compared to oncological dosing (16, 25), presumably due to reduced hyperinflammatory toxicity of low-dose ICIs during acute infection (16). Based on these findings and the known risk of potent proinflammatory immune responses induced by Mucoralean antigens (4, 26, 27), we focused on a low-dose ICI treatment approach.

Based on our rigorous evaluation with 34-36 mice per group tested in 3 independent replicates, low-dose therapy of *R. arrhizus*-infected mice with either anti-PD-1 or anti-PD-L1 significantly reduced morbidity and mortality compared to mock treatment with isotype antibodies. Improvement of morbidity and mortality by day 4 post-infection was comparable in anti-PD-1- and anti-PD-L1-treated animals. However, anti-PD-L1 provided more consistent sustained protection than PD-1 inhibition, as evidenced by an increase in morbidity and mortality of anti-PD-1-treated mice by +7 and greater variation in fungal clearance compared to the anti-PD-L1-treated cohort. Termination of follow-up after 7 days, in order to obtain enough samples from survivors for downstream analyses, might have even underestimated the potential differences in survival outcomes between ICI-treated and isotype-treated cohorts, and between the two ICI treatments. Most mice in the isotype- and anti-PD-1-treated cohorts had day-7 MSS values of ≥2, indicating a high probability of death within the next 1-3 days (21, own unpublished data). In contrast, the combination of low fungal burden and mostly mild infection severity would portend a high probability of survival in the anti-PD-L1-treated cohort.

There are several pharmacological and immunological differences between PD-1 and PD-L1 inhibitors that may have contributed to disparate outcomes. On the one hand, functional *in-vitro* studies with human T cells suggested that PD-L1 inhibitors have greater binding affinity to their target than PD-1-targeted agents, along with superior potency of PD-L1 inhibitors to revert PD-1 axis signaling (28). However, these studies are not immediately transferable to murine models due to the disparate druggability profiles of human and murine checkpoint targets (29).

Unlike anti-PD-L1, PD-1 inhibitors also block the PD-1/PD-L2 interaction (30). PD-L2 upregulation on mononuclear phagocytes is induced by Th2-cytokines and proinflammatory effector cytokines, such as TNF-α or GM-CSF (30, 31). These cytokines are known effectors of the innate immune response to Mucoralean antigens (27, 32, 33) and were strongly enhanced by PD-1 blockade in our IPM model. Consistently, PD-L2 was significantly upregulated on macrophages of anti-PD-1-treated mice. The implications of PD-L2 signaling for immune tolerance to molds and defense against invasive infections are largely unexplored. As both protective and detrimental effects were linked to PD-L2 activation and deficiency in murine sepsis models (34), further data are needed to elucidate the functional impact of the PD-1/PD-L2 interaction and potential PD-1-independent immunomodulatory effects of PD-L2 (35) on the pathogenesis of mucormycosis.

In addition to the upregulation of the PD-1 ligands (PD-L1 and PD-L2) in anti-PD-1-treated mice with IPM, both ICI treatments stimulated Tim-3 expression on macrophages, suggesting compensatory upregulation of alternative checkpoint pathways. This observation is consistent with a clinical report of nivolumab therapy (PD-1 inhibitor) in a patient with intractable mucormycosis and aspergillosis (14). Studies in murine tumor models suggested that compensatory upregulation of co-inhibitory checkpoint pathways can limit the efficacy of single-agent checkpoint blockade (36). However, both the cited case reports and our data in the murine IPM model indicate that initial upregulation of alternative exhaustion markers does not impair therapeutic success. Furthermore, Tim-3 upregulation on macrophages did not correlate with diminished fungicidal activity of *ex-vivo* splenocytes. Nonetheless, it would be essential to thoroughly study the roles of the many alternative checkpoint targets (37) in the immune pathogenesis of IPM and other mold infections in order to determine potential benefits and toxicities (16) of combined checkpoint blockade.

Although not a focus of this study due to the difficulty to reliably assess Th polarization and specific T-cell responses to mold antigens in a background of T-cell-active immunosuppression, especially corticosteroids (38, 39), there were notable differences in Th signature cytokine production in mice with IPM depending on the ICI treatment. While both ICIs modestly enhanced Th2 cytokine production in the lungs of mice with IPM by day +7, only anti-PD-1 elicited a massive early production of Th2 cytokines. Dominance of Th2 responses can interfere with pathogen clearance and portends a poor prognosis in most fungal infections (40). Similarly, although part of the physiological response to Mucoralean antigens (27) and crucial to prevent detrimental hyperinflammation, increased IL-10 production in the lungs of anti-PD-1-treated mice with IPM, indicative of Treg activation, could attenuate fungal elimination (40). However, as we found enhanced pulmonary fungal clearance and fungicidal activity of *ex-vivo* splenocytes in ICI-treated mice with IPM, protective changes to the immune environment (e.g., increased release of neutrophil- and macrophage-attracting chemokines and growth factors) likely outweigh these potentially undesirable effects.

Interestingly, anti-PD-1-treated *R. arrhizus*-infected mice displayed early suppression of pulmonary production of the Th17 signature cytokine IL-17A, whereas PD-L1 blockade slightly increased IL-17A release by day +4. Given the known contribution of Th17 immunity and IL-17A to the clearance of Mucorales and other molds (41, 42), the more favorable IL-17A kinetics in anti-PD-L1-treated versus anti-PD-1-treated mice could be another contributing factor to disparate therapeutic outcomes. Consistent with the results of our previously published IPA study (16), both PD-1 pathway inhibitors did not enhance the release of IFN-γ or other Th1 cytokines in mice with IPM. Therefore, there might be an independent benefit of co-administration of IFN-γ along with ICIs, as suggested by clinical case reports (14, 15).

Lastly, differences in toxicity profiles may have contributed to disparate outcomes between anti-PD-1 and anti-PD-L1-treated mice. While no apparent immunotoxicity was noted after the injections, clinical signs of toxicities can be difficult to distinguish from infection-induced distress and inflammation. Notably, strongly elevated serum concentrations of several proinflammatory cytokines, especially IL-6, were seen in anti-PD-1-treated mice with IPM. IL-6 is increasingly recognized as a key mediator of immunotoxicities in cancer patients receiving ICIs, and IL-6-targeted combination therapy might reduce adverse events (43). Given the considerable number of investigational PD-1/PD-L1-targeted ICI therapeutics currently undergoing evaluation in oncology trials (44), it might also be feasible to identify ICI agents that decouple protective immune enhancement against Mucorales from potential IL-6-mediated toxicities.

This proof-of-concept *in-vivo* study has limitations. On the one hand, this study was based on infection experiments with a single Mucorales species and isolate. Prior *in-vitro* studies in a human alveolar model (45) and murine infection experiments (46) revealed considerable differences in the invasive potential, virulence, and extent of inflammatory host responses between different Mucorales species and isolates. Furthermore, the underlying predisposing conditions and nature of immune dysfunction can considerably impact the course and immunopathology of invasive mucormycosis in mice (46). Although commonly used in translational mycology studies, pharmacological immunosuppression of otherwise healthy mice remains artificial and cannot fully recapitulate the complex and nuanced immune dysfunction caused by the diverse underlying host factors predisposing patients to mucormycosis, such as hematological malignancies, diabetes mellitus, trauma, and COVID-19 (1, 2, 47). Additionally, cancer immunology studies suggested that glucocorticosteroids, which were part of our immunosuppressive regimen, can induce the expression of checkpoint molecules and potentially contribute to ICI resistance (48). Hence, the development of more pathophysiologically relevant mammalian models of invasive mold infections (e.g., murine leukemia models), remains an important pursuit for future immunotherapeutic investigations and in-depth immune profiling efforts (49). Furthermore, while our results document a considerable mono-therapeutic benefit of PD-1/PD-L1 axis blockade in mice with IPM, synergistic activity of ICIs with Mucorales-active antifungal agents needs to be studied, especially those with known immunomodulatory properties, such as liposomal amphotericin B or even the echinocandins (50). Lastly, more dynamic assessment of immune cell activation and recruitment would be warranted, for example, by using non-lethal tracer-guided tracking of neutrophils or mononuclear phagocytes (51).

In summary, in this study, we provided, for the first time, a direct comparison of PD-1 and PD-L1 blocking antibodies to treat IPM in a mammalian *in-vivo* model. Both ICI treatments strongly improved morbidity and mortality of *R. arrhizus*-infected mice, adding to an increasing body of evidence pointing to the PD-1 immune checkpoint pathway as an appealing therapeutic target for opportunistic infections (12). Furthermore, our results hint at nuanced and complex immunological responses to PD-1 versus PD-L1 blockade in mice with IPM, with the latter providing an overall more favorable balance between protective immune enhancement and surrogates of potential immunotoxicity. Development of more pathophysiologically representative preclinical models of invasive mold infections, deployment of the enormous potential of “omics” technologies to thoroughly study the pulmonary immune microenvironment in ICI-treated mice, and dose-response studies will be essential in order to fully define and eventually exploit the “sweet spot” between ICI-mediated augmentation of anti-mold defense and the risk of overzealous immune injury.

## Data Availability Statement

The original contributions presented in the study are included in the article/**Supplementary Material**. Further inquiries can be directed to the corresponding authors.

## Ethics Statement

The animal study was reviewed and approved by MD Anderson Cancer Center Institutional Animal Care and Use Committee.

## Author Contributions

SW and DK conceived the study and designed experiments. SW, NA, and UB performed experiments. SW, UB, MK, JT, ND, and DK analyzed and interpreted data. SW visualized data. SW and DK wrote the paper. All authors provided revisions and approved the final version of the manuscript prior to submission.

## Funding

This study was funded by the MD Anderson Cancer Center, Division of Internal Medicine Research and Quality Improvement Award (to SW). Parts of this study were further supported by the Robert C. Hickey Chair for Clinical Care endowment (to DK). Flow cytometric analyses were performed in collaboration with the MD Anderson Cancer Center Flow Cytometry and Cellular Imaging Core Facility, which is supported in part by the National Institutes of Health through MD Anderson’s Cancer Center Support Grant CA016672.

## Conflict of Interest

DK reports honoraria and research support from Gilead Sciences and Astellas Pharma. He received consultant fees from Astellas Pharma, Merck, and Gilead Sciences, and is a member of the Data Review Committee of Cidara Therapeutics, AbbVie, Scynexis, and the Mycoses Study Group.

The remaining authors declare that the research was conducted in the absence of any commercial or financial relationships that could be construed as a potential conflict of interest.

## Publisher’s Note

All claims expressed in this article are solely those of the authors and do not necessarily represent those of their affiliated organizations, or those of the publisher, the editors and the reviewers. Any product that may be evaluated in this article, or claim that may be made by its manufacturer, is not guaranteed or endorsed by the publisher.
