# Supplementary material for: Blockade of the PD-1/PD-L1 Immune Checkpoint Pathway Improves Infection Outcomes and Enhances Fungicidal Host Defense in a Murine Model of Invasive Pulmonary Mucormycosis
Source: Front Immunol. 2022 Feb 18;13:838344. doi: 10.3389/fimmu.2022.838344 (PMC8896628; doi:10.3389/fimmu.2022.838344)
Supplement: Supplementary file 1 [file DataSheet_1.docx]

Blockade of the PD-1/PD-L1 immune checkpoint pathway

improves infection outcomes and enhances fungicidal host

defense in a murine model of invasive pulmonary mucormycosis

Supplementary Material

**Supplementary Figure 1.** **Pulmonary cytokine and chemokine concentrations on day +4.**

Cytokine and chemokine concentrations in lung tissue homogenates from *R. arrhizus*-infected mice receiving ICIs or isotype antibodies were measured on day +4 using a 19-plex Luminex assay. N = 5 mice per treatment arm. In addition to individual values, median lung concentrations per treatment arm (rounded to 1 pg/g) and median-to-median ratios (MMR) are provided. The two-sided Mann-Whitney U test with Benjamini-Hochberg adjustment for a false discovery rate of 0.2 was used for significance testing (ICI-treated mice versus controls and comparisons between the two ICI treatments). C-reactive protein is not displayed (>100 ng/g in all samples, distributions not significantly different among the four groups). Abbreviations: C(X)CL = C(-X)-C motive chemokine receptor ligand, (G)M-CSF = (Granulocyte) Macrophage Colony-Stimulating Factor, IFN = Interferon, IL = Interleukin, MCP = Monocyte Chemoattractant Protein, MIP = Macrophage Inflammatory Protein, RANTES = Regulated upon Activation, Normal T cell Expressed and Presumably Secreted, TNF = Tumor Necrosis Factor.

**Supplementary Figure 2.** **Pulmonary cytokine and chemokine concentrations on day +7.**

Cytokine and chemokine concentrations in lung tissue homogenates from *R. arrhizus*-infected mice receiving ICIs or isotype antibodies were measured on day +7 using a 19-plex Luminex assay. N = 5 mice per treatment arm. In addition to individual values, median lung concentrations per treatment arm (rounded to 1 pg/g) and median-to-median ratios (MMR) are provided. The two-sided Mann-Whitney U test with Benjamini-Hochberg adjustment for a false discovery rate of 0.2 was used for significance testing (ICI-treated mice versus controls and comparisons between the two ICI treatments). C-reactive protein is not displayed (>100 ng/g in all samples but one, distributions not significantly different among the four groups). Abbreviations: C(X)CL = C(-X)-C motive chemokine receptor ligand, (G)M-CSF = (Granulocyte) Macrophage Colony-Stimulating Factor, IFN = Interferon, IL = Interleukin, MCP = Monocyte Chemoattractant Protein, MIP = Macrophage Inflammatory Protein, RANTES = Regulated upon Activation, Normal T cell Expressed and Presumably Secreted, TNF = Tumor Necrosis Factor.

**Supplementary Figure 3.** **Correlation of exhaustion marker expression and fungal killing by murine splenocytes.** Correlation plots comparing the percentages of PD-1-expressing T cells (CD3^+^) and CTLA-4-expressing NK cells (CD49b^+^) with mycelial expansion of *R. arrhizus* in co-cultures with splenocytes from the same mice. The latter was tested at three different effector/target (E:T) ratios, as described in Materials & Methods. Spearman’s rank correlation coefficients (ρ) and their p-value are provided. N = 20. Abbreviations: CD = Cluster of Differentiation, CTLA-4 = Cytotoxic T-Lymphocyte-Associated Protein 4, Norm. = normalized (to ‘fungus only’ control without splenocytes), PD-1 = Programmed Cell Death Protein 1.

**Supplementary Figure 4.** **Serum cytokine and chemokine concentrations on day +4.**

Serum cytokine and chemokine concentrations of *R. arrhizus*-infected mice receiving ICIs or isotype antibodies were measured on day +4 using a 19-plex Luminex assay. N = 5 mice per treatment arm. In addition to individual values, median lung concentrations per treatment arm (rounded to 0.1 pg/mL) and median-to-median ratios (MMR) are provided. The two-sided Mann-Whitney U test with Benjamini-Hochberg adjustment for a false discovery rate of 0.2 was used for significance testing (ICI-treated mice versus controls and comparisons between the two ICI treatments). C-reactive protein is not displayed (>100 ng/g in all samples, distributions not significantly different among the four groups). Abbreviations: C(X)CL = C(-X)-C motive chemokine receptor ligand, (G)M-CSF = (Granulocyte) Macrophage Colony-Stimulating Factor, IFN = Interferon, IL = Interleukin, MCP = Monocyte Chemoattractant Protein, MIP = Macrophage Inflammatory Protein, RANTES = Regulated upon Activation, Normal T cell Expressed and Presumably Secreted, TNF = Tumor Necrosis Factor.

**Supplementary Figure 5.** **Serum cytokine and chemokine concentrations on day +7.**

Serum cytokine and chemokine concentrations of *R. arrhizus*-infected mice receiving ICIs or isotype antibodies were measured on day +7 using a 19-plex Luminex assay. N = 6-9 mice per treatment arm. In addition to individual values, median lung concentrations per treatment arm (rounded to 0.1 pg/mL) and median-to-median ratios (MMR) are provided. The two-sided Mann-Whitney U test with Benjamini-Hochberg adjustment for a false discovery rate of 0.2 was used for significance testing (ICI-treated mice versus controls and comparisons between the two ICI treatments). C-reactive protein is not displayed (>100 ng/g in all samples, distributions not significantly different among the four groups). Abbreviations: C(X)CL = C(-X)-C motive chemokine receptor ligand, (G)M-CSF = (Granulocyte) Macrophage Colony-Stimulating Factor, IFN = Interferon, IL = Interleukin, MCP = Monocyte Chemoattractant Protein, MIP = Macrophage Inflammatory Protein, RANTES = Regulated upon Activation, Normal T cell Expressed and Presumably Secreted, TNF = Tumor Necrosis Factor.
